# A Versatile Hemolin With Pattern Recognitional Contributions to the Humoral Immune Responses of the Chinese Oak Silkworm *Antheraea pernyi*

**DOI:** 10.3389/fimmu.2022.904862

**Published:** 2022-05-20

**Authors:** Xueshan He, Tianyang Zhou, Yuchen Cai, Yang Liu, Siqi Zhao, Jinghai Zhang, Xialu Wang, Rong Zhang

**Affiliations:** ^1^School of Medical Devices, Shenyang Pharmaceutical University, Shenyang, China; ^2^School of Life Science and Bio-Pharmaceutics, Shenyang Pharmaceutical University, Shenyang, China; ^3^Research and Development Department, Liaoning Applos Biotechnology Co., Ltd, Shenyang, China

**Keywords:** pattern recognition receptor, hemolin, humoral immunity, prophenoloxidase activating system, antimicrobial peptide synthesis

## Abstract

Hemolin is a distinctive immunoglobulin superfamily member involved in invertebrate immune events. Although it is believed that hemolin regulates hemocyte phagocytosis and microbial agglutination in insects, little is known about its contribution to the humoral immune system. In the present study, we focused on hemolin in *Antheraea pernyi* (*Ap*-hemolin) by studying its pattern recognition property and humoral immune functions. Tissue distribution analysis demonstrated the mRNA level of *Ap*-hemolin was extremely immune-inducible in different tissues. The results of western blotting and biolayer interferometry showed recombinant *Ap*-hemolin bound to various microbes and pathogen-associated molecular patterns. In further immune functional studies, it was detected that knockdown of hemolin regulated the expression level of antimicrobial peptide genes and decreased prophenoloxidase activation in the *A. pernyi* hemolymph stimulated by microbial invaders. Together, these data suggest that hemolin is a multifunctional pattern recognition receptor that plays critical roles in the humoral immune responses of *A. pernyi*.

## Introduction

It is a common notion that discrimination between the self and infectious invaders is essential for the initiation of an immune response. In vertebrates, it depends on diverse antibodies, the representative members of the immunoglobulin superfamily (IgSF) that distinguish between the self and nonself (1, 2). In vertebrates, it is the limited germline-encoded pattern recognition receptors (PRRs) that exercise the function of recognizing pathogen-associated molecular patterns (PAMPs), which are a set of limited, highly conserved surface molecular structures of pathogens that are absent from host cells (3, 4). After infectious nonself recognition, two branches of innate immune systems, cellular and humoral immune responses, are triggered in insects to eliminate invaders (5). Cellular responses recruit various hemocytes, leading to phagocytosis, encapsulation and nodulation of microbes (6). Humoral responses include synthesis of antimicrobial peptides (AMPs) and the activation of the prophenoloxidase (PPO) activating system, resulting in melanization of invaders (7–9).

Hemolin is thus a unique invertebrate-derived immunoglobulin superfamily member that participates in immune events. It was first identified in the bacterial-induced pupal hemolymph of *Hyalophora cecropia* as an important component in the acute phase humoral immune defense protein complex that binds to the bacterial surface (10). A highly homologous cDNA was isolated and sequenced as hemolin (previously named P4) from the larvae of *Manduca sexta*. In addition to binding to bacterial cells, hemolin in *M. sexta* also associates with the surface of hemocytes to inhibit hemocyte aggregation (11). Further investigations of the deduced amino acid sequence and crystal structure reveal that hemolin in *H. cecropia* has a close relation to insect and vertebrate neural adhesion molecules and arranges four internal repeated C2-type Ig domains into a strongly bent horseshoe structure (10, 12). Over the past few decades, numerous hemolin homologs have been identified, and their expression properties have been well studied in insects (13–19). The mRNA expression of insect hemolins is found in several tissues over almost all stages of development, in which the expression level intriguingly increases during metamorphosis, diapause (20) and molting periods (21–24). Furthermore, the expression level is immune-inducible and presents at aconstitutively low level in native samples but increases dramatically after pathogen injection. In view of the structure and expression studies, we can be confident that hemolin has a significantly protective role in the whole life cycle of insects. In 2015, it came as a surprise to find a hemolin-like protein identified from *Litopenaeus vannamei*, breaking the view that hemolin is only expressed in lepidopterans (25).

The intriguing fact that hemolin is a bacteria-inducible protein but lacks direct antibacterial ability has encouraged many attempts to understand its immune functions. Several studies have demonstrated that hemolin acts as a PRR with broad specificity to bacterial strains and fungi, causing them to aggregate (10, 26, 27). Available data show that the binding of hemolin to bacterial surfaces occurs mainly through its interaction with the Lipid A moiety of lipopolysaccharide (LPS) from Gram-negative bacteria and lipoteichoic acid (LTA) from Gram-positive bacteria (12, 27, 28). Although there are various indications that hemolin might bind to peptidoglycan and β-1,3-glucan, there is no conclusive evidence for the binding of hemolin to these PAMPs.

In addition to the nonspecifically binding capacity to hemocytes and modulating hemocyte aggregation and phagocytosis (26, 29–31), hemolin is involved in opsonizing nonself targets for phagocytosis and encapsulation (29, 32). There is now evidence that hemolin is involved in lepidopteran melanization. RNA interference with hemolin causes depletion of phenoloxidase activity in *H. cecropia* pupae, but there was no significant interference with the induction of antimicrobial peptides (33). This study provided clues that hemolin is also probably involved in the regulation of insect humoral immune responses. Thus, further experiments are required to more completely characterize the pattern recognition properties of hemolin and to determine what kinds of immune responses it takes part in.

The present study is an attempt to reach some understanding of hemolin in *A. pernyi* regarding its pattern recognition properties and exact function in the humoral immune system. We investigated the binding affinity of *Antheraea pernyi* hemolin-like protein (abbreviated as *Ap*-hemolin in this paper) to typical microorganisms and PAMPs. Further humoral immune functional investigation focused on the regulation of antimicrobial peptide expression and hemolymph PPO activation by *Ap*-hemolin in response to microbial stimulators. The results suggest that *Ap*-hemolin probably serves as a versatile pattern recognition receptor and plays crucial roles in the diverse humoral immunity of *Antheraea pernyi.*

## Materials and Methods

### Insects, Microorganisms and PAMPs

*A. pernyi* larvae were purchased from Shenyang Agricultural University and reared on a natural diet at 23 ± 2°C. The bacterial strains and fungi involved in the experiments were provided by another laboratory, including G^-^ bacteria *Escherichia coli* (CMCC44102) and *Pseudomonas aeruginosa* (CMCC10104), G^+^ bacteria *Micrococcus luteus* (CMCC28001) and *Staphylococcus aureus* (CMCC26003), and fungi *Candida albicans* (CMCC98001) and *Saccharomyces cerevisiae* (S2061).

PAMPs containing LPS (from *E. coli* O55:B5, L2880), laminarin (from *L. digitata*, L9634), LTA (from *B. subtilis*, L3265), Lys-PGN (from *M. luteus*, 53243), DAP-PGN (from *B. subtilis*, 69554), and mannan (from *S. cerevisiae*, M7504) were purchased from Sigma–Aldrich (Poole, USA), and curdlan (insoluble β-1, 3 glucan) was purchased from Wako Pure Chemicals (Osaka, Japan).

### Recombinant His_6_-Ap-Hemolin and Antibody Preparation

A gene fragment encoding mature *Ap*-hemolin (GenBank ID: KF938917.1) was inserted into a pSYPU-1b expression vector constructed by our laboratory at the restriction enzyme sites for *Nco I* and *EcoR I* (TaKaRa, China), followed by transformation into *E. coli* (strain *DE3*). Following sequencing, the positive colonies were inoculated into LB medium containing 50 μg/ml kanamycin and cultured at 37°C with 200 rpm of agitation. In the mid-logarithmic phase of culture growth, the overexpression of *Ap*-hemolin with a His-tag at the amino terminus was induced by the addition of IPTG to a final concentration of 0.2 mM. For purification, the bacteria were harvested by centrifugation at 3500 × g for 20 min. The pellet was resuspended in buffer A (20 mM Tris-HCl, 500 mM NaCl, pH 7.0) and sonicated in an ultrasonic oscillator. After sonication, the supernatant of the sonicated sample was collected by centrifugation at 12000 × g for 20 min. Then, the supernatant was loaded into a Ni-Sepharose column (GE Healthcare) equilibrated with buffer A and eluted with a linear gradient of 10-500 mM imidazole in buffer A. The fractions containing His_6_-Ap-hemolin were pooled and dialyzed against buffer B (20 mM Tris-HCl, pH 7.0). A rabbit antiserum was raised against recombinant His_6_-*Ap*-hemolin, and the polyclonal antibody was purified using HiTrap™ MabSelect SuRe media (GE Healthcare) according to the manufacturer’s instructions.

### Expression of Ap-Hemolin in Response to Pathogen Injection

For immune challenge, fifth-instar larvae were subjected to an injection (20-μl volume) of mixed microorganism (*S. aureus*, *E. coli* and *C. albicans*) suspension diluted with insect saline at a final concentration of 1 × 10^8^ CFU/ml. Subsequently, larvae were anesthetized on ice and dissected for tissues, including the fat body, midgut, epidermis, Malpighian tube and hemocytes, at different time points. Larvae injected with the same volume of insect saline were used as negative controls. Total RNA was extracted from each tissue sample with TRIzol reagent (Thermo Scientific™, USA) followed by reverse transcription with a RevertAid First Stand cDNA Synthesis Kit (Thermo Scientific™, USA) according to the manufacturer’s instructions. Taking the synthesized cDNA as a template, quantitative real-time PCR (qPCR) was performed to examine the level of *Ap*-hemolin mRNA expression in diverse tissues in response to immune injection. For qPCR, a 140-bp fragment of *Ap*-hemolin was amplified with a CFX96 Optics Module (Bio-Rad); and cycling conditions were as follows: 95°C for 120 s, followed by 50 cycles of 95°C for 15 s and 60°C for 30 s with a final extension of 72°C for 10 min. After amplification, the relative expression level was calculated using the 2^-ΔΔCt^ method, which employed the 18S rRNA gene as an endogenous control. The primers used in all experiments are listed in **Table 1**.

**Table 1:** Sequence information of primers used in the study.

Target gene	Primer name	Sequence (5’-3’)	
**Primers used for hemolin cDNA cloning**			
*Hemolin*	Forward		CAGCCCGTGGAGAAGCTGCCGGT
	Reverse		CTAATTAACTTGGACCAAGGTCTCGACGTATTCGT
**Primers used for dsRNA synthesis**			
*Hemolin*	Forward		TAATACGACTCACTATAGGGTTGCAAGAACATAACGTTGCCC
	Reverse		TAATACGACTCACTATAGGGGATGTACTGTGGAACAGGCTCACC
*EGFP*	Forward		TAATACGACTCACTATAGGGGCAAGGGTGAGGAACT
	Reverse		TAATACGACTCACTATAGGGACAGCTCGTCCATGCCG
**Primers used for Real-time PCR**			
*Hemolin*	Forward		TATGATGGCGAAGGCTGGT
	Reverse		AGGTTCTATTGTGGCGGGTG
*Defensin*	Forward		TAACCATCAGCGGCAATA
	Reverse		GTTCTCCACAGTCCAAGA
*Attacin*	Forward		TGGATTGGCTTATGATAATGTC
	Reverse		GGTTGTCGTTGTGGAATAG
*Moricin*	Forward		ATCGGATGTTAATATACAGTAAGT
	Reverse		ACACAATAAAGCACAGCAA
*Spätzle*	Forward		AAATTGGGCTTCTGCGAAT
	Reverse		TCTGGTGTGTCAGGTAAATCCA
*Fadd*	Forward		TCAGAATACTCTCAACTTAA
	Reverse		GAGGTGCTACATTATCTTC
*18S rRNA*	Forward		CGATCCGCCGACGTTACTAC
	Reverse		GTCCGGGCCTGGTGAGATT

T7 promoter sequences were underlined.

Western blotting assays were performed to detect the amount of hemolin protein in the *A. pernyi* hemolymph upon microbe injection. Fifth-instar larvae were subjected separately to an injection (20 μl volume) with different microorganism (1 × 10^8^ CFU/ml *S. aureus*, *E. coli* and *C. albicans*) suspensions diluted with insect saline. Twelve hours after injection, the cell-free plasma samples were collected as described by Wen et al. (34). Then, 125 micrograms of total protein in each plasma sample were collected and subjected to 12% SDS–PAGE, followed by transfer to PVDF membranes (Millipore, USA). The membranes were blocked with 5% skim milk in TBST and incubated with the primary rabbit anti-His_6_-*Ap*-hemolin polyclonal antibody (1:8,000 diluted) for 1.5 hr. Antibody binding was visualized by a color reaction catalyzed by horseradish peroxidase (HRP) conjugated to goat anti-rabbit IgG (1:8,000 diluted).

### Western Blotting and Biolayer Interferometry Binding Assay

Western blotting assays were conducted to evaluate the binding ability of *Ap*-hemolin to formaldehyde-fixed microbial cells and insoluble PAMPs. Briefly, 200 μl of His_6_-*Ap*-hemolin (500 ng/μl in 20 mM Tris-HCl pH 7.0) was incubated with each set of 100 μl of microorganisms (or insoluble PAMPs) at 37°C for 30 min. After washing with 1 M NaCl and 20 mM Tris-HCl at pH 7.0 three times and elution (2% SDS, 20 mM Tris-HCl, pH 7.0), the bound samples were collected and subjected to 12% SDS–PAGE, followed by immunoblotting as described in Section 2.3.

Afterward, the binding affinity of *Ap*-hemolin to six different soluble PAMPs was analyzed by biolayer interferometry using the Octet K2 system (ForteBio, USA). All experiments were performed at 25°C in a buffer containing 20 mM Tris-HCl at pH 7.0. Nickel-nitrilotriacetic acid (Ni-NTA) biosensors were pre-equilibrated in buffer for at least 10 min before use. Then, recombinant His_6_-*Ap*-hemolin with a His-tag (20 ng/μl, 20 mM Tris-HCl, pH 7.0) was loaded onto Ni-NTA biosensors for 300 s for immobilization, and the binding properties were monitored by flowing serially diluted PAMPs (50~150 μg/ml) through the system. The interference patterns from the protein with buffer and uncoated biosensors with PAMP at the corresponding concentrations were measured as two sets of controls. We used double-reference corrections to subtract the binding signals, and the binding constant values were estimated with the corrected data using Octet data analysis software.

### RNA Interference and the Effect on the Synthesis of Antimicrobial Peptides

Second instar larvae were used for all RNA interference (RNAi) experiments. For dsRNA preparation, DNA fragments encoding hemolin in the *A. pernyi* and EGFP genes were amplified with T7 promoter sequences in reverse orientation, and dsRNA synthesis was performed using a TranscriptAid T7 High Yield Transcription Kit (Thermo Scientific) according to the manufacturer’s instructions. Double-stranded RNA was solubilized in insect saline to a final concentration of 40 ng/μl prior to injection. For RNAi, 5 μl of dsRNA was injected into the hemocoel of 2^nd^ instar larvae (5 individuals for each set) using a sterile microsyringe. The whole body was collected after rearing for additional 12- and 24-hr post-dsRNA treatments, and the knockdown efficiency was evaluated by qPCR and western blotting assays.

Immune challenge was performed by injecting different microorganisms (*S. aureus*, *E. coli* and *C. albicans*). Briefly, 5 μl of microbial cells (2 × 10^8^ CFU/ml in PBS) was injected into the RNAi-treated larvae. Total RNA was isolated from the whole body 12 hr later followed by cDNA synthesis, and qPCR was then performed to detect the relative expression of selected antimicrobial peptides together with the factors of AMP synthesis signaling pathways, including *defensin*, *attacin*, *moricin, spätzle* and *Fas-associating protein with a novel death domain (Fadd)* as previously described in Section 2.3.

### PO Activity Assay

As described above, approximately 200 ng (5 μl, 40 ng/μl) of dsRNA was injected into the hemocoel of each larva. Following rearing for an additional 24 hr, cell-free plasma was collected (20 individuals for each set). The PO assay was carried out as previously described (35). Briefly, an aliquot of 10 μl of cell-free hemolin deficiency plasma was incubated with 10 μl of microbes or PAMPs solution at 25°C for 5 min. Plasma incubated with pathogen solvent was employed as a control. After incubation, 470 μl of substrate solution (1 mM 4-methylcatechol, 2 mM 4-hychoxyproline ethyl ester in 20 mM Tris-HCl, pH 7.0) was added to give a final volume of 500 μl. The sample was maintained at 25°C, and the absorbance was continuously monitored at 520 nm using a microplate reader (Thermo Scientific). One unit of PO activity was calculated as a 0.01 increase in A_520_ per minute.

In addition, the role of recombinant His_6_-*Ap*-hemolin on proPO activation was investigated as previously described (36). Briefly, 10 μl of native hemolymph was preincubated with 10 μl of rHis_6_-*Ap*-hemolin (20 ng/μl) and 10 μl of microbes (5× 10^7^ CFU/ml, 20 mM Tris-HCl, pH 7.0) or PAMPs (20 mM Tris-HCl, pH 7.0) at 25°C for 5 min, and 480 μl of substrate solution was added. Each set was continuously incubated at 25°C, and the absorbance was monitored at 520 nm as described. As negative controls, samples without rHis_6_-*Ap*-hemolin, PAMPs (or microbes) or both (replaced by an appropriate buffer) were examined. Similarly, the role of endogenous hemolin protein on proPO activation was investigated. An aliquot of 10 ml purified anti-His_6_-*Ap*-hemolin antibody (0.8 mg/ml) was pre-incubated with 90 ml native hemolymph at 25°C for 30 min for generation of hemolin deficiency plasma. Hemolymph pre-incubated with the same volume of buffer (20 mM Tris-HCl, pH7.0) was used as the negative control. Then the PO activity stimulated by different elicitors (microbes and PAMPs) was examined and compared as above.

### Statistical Analysis

All experiments were performed at least three times, and similar results were obtained. Each bar represents the mean ± SD (n=3). The results of the effect of hemolin on the synthesis of antimicrobial peptides and PO activity assays were subjected to analysis of variance, followed by Tukey’s multiple comparisons tests.

## Results

### Preparation of Recombinant His_6_-Ap-Hemolin and Its Antibody

**Figure 1:**
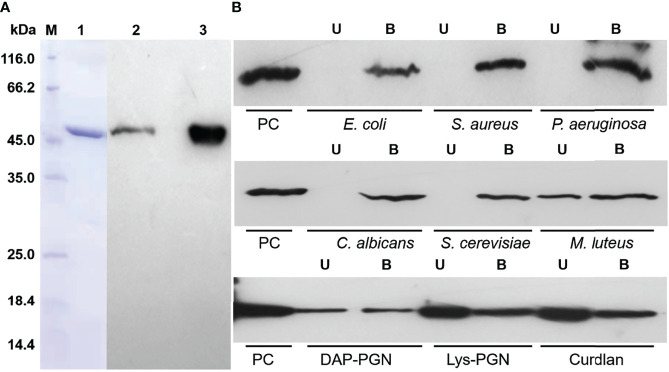
Identification of His_6_-*Ap*-hemolin and its binding to insoluble pathogens. **(A)** SDS–PAGE and immunoblot analysis of *Ap*-hemolin. Lane 1: purified recombinant His_6_-*Ap*-hemolin (5 μg, Coomassie Brilliant Blue staining); Lane 2: natural hemolin in *A. pernyi* hemolymph (5 mg/ml); Lane 3: His_6_-*Ap*-hemolin (40 μg/ml); both Lanes 2 and 3 (30 μl volume per lane) were detected by western blotting assay using the anti-*Ap*-hemolin antibody. **(B)** Binding of His_6_-*Ap*-hemolin to microorganisms and insoluble PAMPs was detected by western blotting. A total of 10 μg of His_6_-*Ap*-hemolin was incubated with microbial cells or insoluble PAMPs. Supernatant and washing fractions were combined as the unbound sample (U). The bound sample **(B)** was eluted with 1 × SDS sample buffer. His_6_-*Ap*-hemolin was used as a positive control (PC). Three biological replicates were used.

To further investigate the role of hemolin in *A. pernyi* larval immune responses, recombinant His_6_-*Ap*-hemolin produced in a prokaryotic expression system was purified with nickel affinity chromatography (**Figure 1**). As shown in **Figure 1A**, the purified soluble protein migrated as a single band of approximately 48 kDa (Lane 1, 5 μg His_6_-*Ap*-hemolin), which was used as an antigen for subsequent production of a polyclonal rabbit antiserum against *Ap*-hemolin. To detect the existence and amount of natural hemolin protein in the *A. pernyi* larval hemolymph, a western blotting assay was performed with the anti-*Ap*-hemolin antibody purified from rabbit antiserum. Through the gray-scale statistical analysis, a trace amount of natural hemolin (~0.3345 μg) was detected in the hemolymph of native larvae (**Lane 2,** approximately 150 μg of protein in total), the molecular mass of which was approximately the same as that of the recombinant protein (**Lane 3,** 1.2 μg His_6_-*Ap*-hemolin).

### Expression Profiles of Ap-Hemolin in Response to Microbial Stimulation

**Figure 2:**
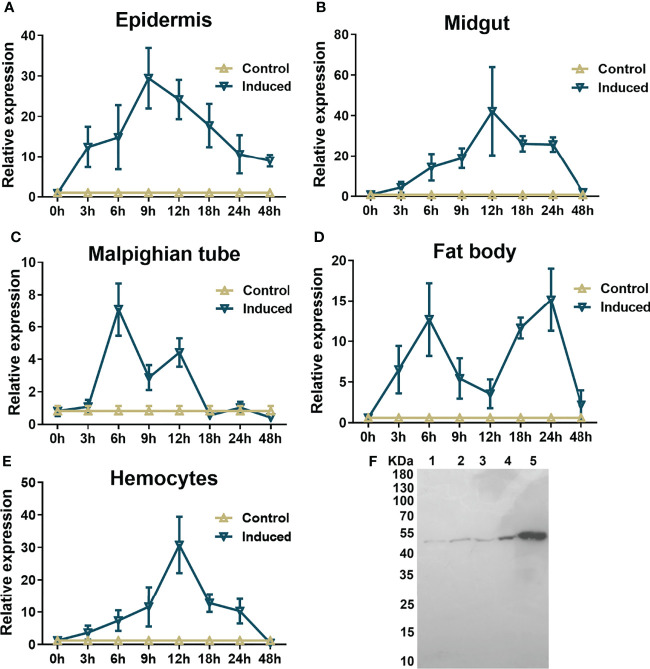
Expression profiles of hemolin in *A. pernyi* larvae. Tissue distribution analysis of *Ap*-hemolin mRNA in response to immune challenge was detected at different immune challenge times (0, 3, 6, 9, 12, 18, 24 and 48 hr). Tissues involved in the experiment were epidermis **(A)**, midgut **(B)**, Malpighian tube **(C)**, fat body **(D)** and hemocytes **(E)**. Each tissue was collected at different times from the samples (five individuals per time point) injected with insect saline (Control) or the ones immune challenged with the mixture of *S. aureus, E. coli* and *C. albicans* (Induced). Each bar represents the mean ± SD (N=3). **(F)** Changes in hemolin protein levels in cell-free plasma upon microbe injection. Samples were collected before injection (NT) or 12 h after challenge with microbe injection. Lane 1: nontreated 5^th^ instar larvae plasma (NT); Lane 2: larvae plasma stimulated by *S. aureus*; Lane 3: larvae plasma stimulated by *C. albicans*; Lane 4: larvae plasma stimulated by *E. coli*; Lane 5: recombinant His_6_-*Ap*-hemolin (2.25 μg, a marker to indicate the migration of endogenous hemolin in hemolymph). All lanes were detected by western blotting assay using anti-His_6_-*Ap*-hemolin antibody (total protein in lane 1-4: 125 μg). Three biological replicates were used.

Significant upregulation of mRNA expression was observed in all examined immune-challenged tissues, including the epidermis, fat body, hemocytes, Malpighian tube and midgut, in comparison to those injected with the same amount of insect saline instead of formaldehyde-killed microorganisms 3 hr after injection (**Figure 2**). However, the changes in different tissues in response to stimulation varied within 48 hr post injection. After microbial injection, the mRNA level of hemolin in the epidermis increased significantly within the first 3 hr and reached a peak at 9 hr. Although the mRNA level of hemolin rapidly declined after the peak, it maintained a high level at 48 hr post-stimulation, approximately ten fold higher than that without immune challenge at the same time point **(** **Figure 2A****)**. The same trend was detected in both the midgut and hemocytes, except that the level peaked at 12 hr after microbial injection and the level finally decreased to baseline at 48 hr post-injection (**Figures 2B, E**). Interestingly, in the case of the Malpighian tube and fat body, there were two mRNA level peaks. As shown in **Figure 2C**, the mRNA level in the Malpighian tube barely changed in the first 3 hr post-stimulation but rose sharply to the peak at 6 hr post-injection. After a rapid fall into the trough, the second expression peak appeared at 12 hr, and the level dropped into the baseline at 18 hr. In the fat body, after hitting the first peak value at 6 hr post-injection, the level slipped near the base level at 12 hr and then increased rapidly to the second expression soar at 24 hr post-injection **(** **Figure 2D****)**. Several studies have demonstrated the involvement of secreted hemolin in the early humoral immune response. To investigate the probable roles of hemolin in humoral immunity, its protein distribution in the larval hemolymph of *A. pernyi* upon microbe stimulation was evaluated by western blotting. Compared with that in the untreated group, the amount of hemolin in the immune-challenged groups was significantly upregulated (**Figure 2F**). At 12 h after microbe injection, the amount of hemolin increased more in *E. coli- and* S. *aureus*-injected samples (approximately 1.5- and 1.0-fold increases) than in the untreated sample, while the level of protein upregulation was lower upon fungus *C. albicans* stimulation (~0.5-fold). These results indicate that the expression of hemolin is sensitive to different microbial invaders.

### Pattern Recognition Properties of Ap-Hemolin

To date, previous studies have confirmed that hemolin is the only IgSF member considered a PRR that binds diverse microbes and that hemolin in *M. sexta* interacts with bacteria *via* the cell wall-derived PAMPs LPS and LTA (27). However, we still do not know how hemolin binds to fungal cells and whether it associates with other components on the surfaces of bacterial cells. Our current research is focused on the pattern recognition properties of *Ap*-hemolin. First, its binding capacity to pathogens was detected by western blotting assay with the recombinant hemolin protein. As shown in **Figure 1B**, valid binding of His_6_-*Ap*-hemolin to each microbial cell was observed, including G^-^ bacteria *E. coli* and *P. aeruginosa*, G^+^ bacteria *M. luteus* and *S. aureus*, and fungi *C. albicans* and *S. cerevisiae*. In comparison, hemolin appeared in the unbound sample of *M. luteus*, revealing the relatively weaker binding ability of hemolin to this kind of microorganism. Further investigations of the molecular interaction between His_6_-*Ap*-hemolin and microbial cell wall-derived PAMPs were carried out to gain some understanding of its binding properties. Recombinant *Ap*-hemolin was detected to have a broad-spectrum binding capacity of insoluble PAMPs, but in the same incubation amount of His_6_-*Ap*-hemolin and insoluble PAMPs, the amount of binding fractions was distinct, suggesting different binding abilities of *Ap*-hemolin to diverse pathogens.

**Figure 3:**
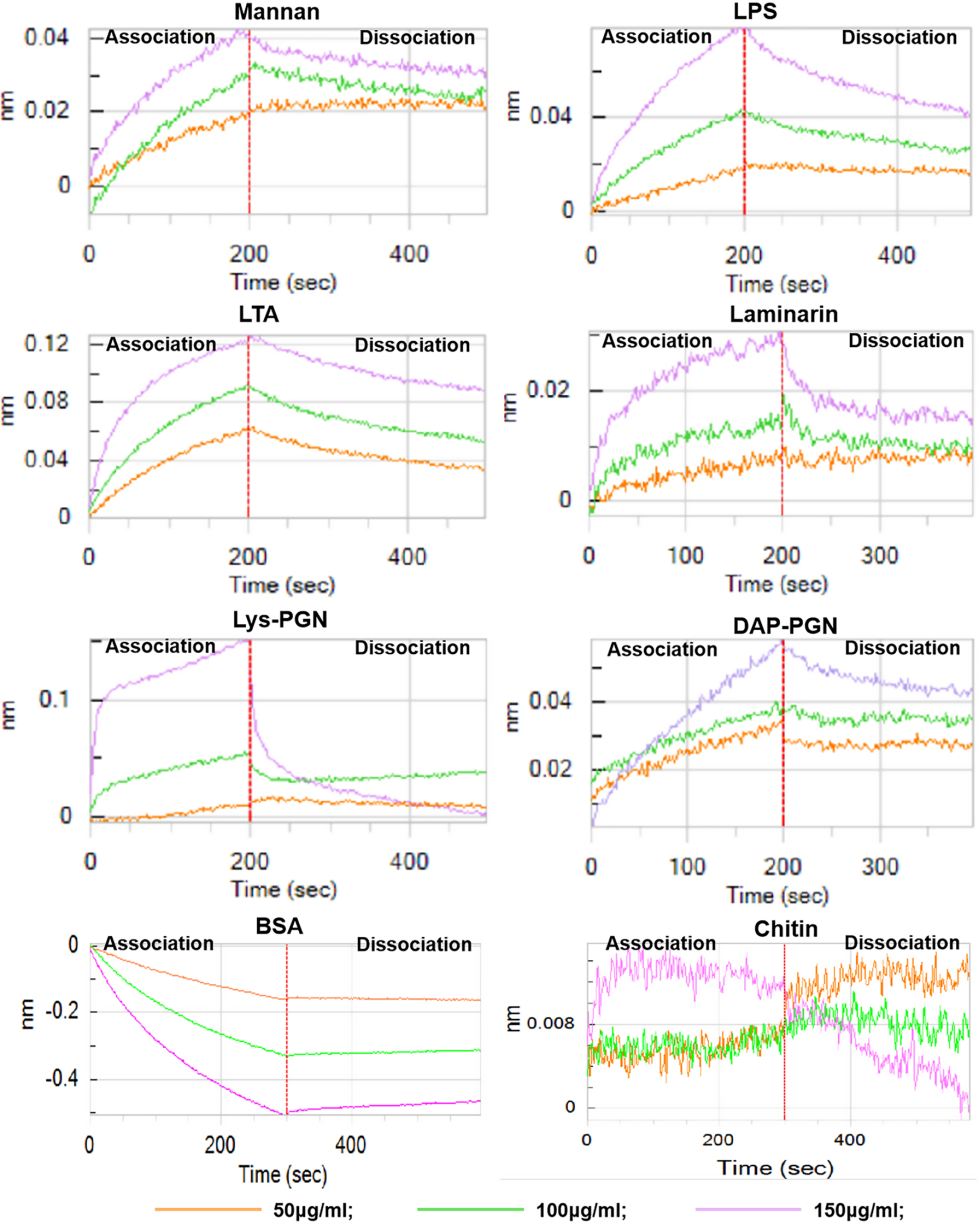
Binding of His_6_-*Ap*-hemolin to soluble PAMPs. A biolayer interferometry binding assay was performed to detect the binding of His_6_-*Ap*-hemolin to six soluble microbe-derived PAMPs, including mannan, LPS, LTA, laminarin, Lys-PGN and DAP-PGN. The vertical and horizontal axes represent the light shift distance (nm) and association/dissociation time (s), respectively. As negative controls, the binding of His_6_-*Ap*-hemolin to chitin (C9752) and bovine serum albumin (V900933) purchased from Sigma–Aldrich (Poole, USA) was also examined.

Furthermore, octet biolayer interferometry was employed to evaluate the binding affinity of His_6_-*Ap*-hemolin to microbial PAMPs in solvable conditions (**Figure 3**). The results validated that *Ap*-hemolin also binds to soluble PAMPs, including laminarin (soluble β-1,3-glucan), Lys-PGN (lysine-type peptidoglycan), DAP-PGN (mesodiaminopimelic acid-type peptidoglycan), LPS, LTA and mannan, almost in a concentration-dependent manner. Although the precise association and dissociation constants could not be calculated due to the lack of a definite molecular weight obtained from the mixed PAMPs, the trend of association and dissociation curves shows that His_6_-*Ap*-hemolin has broad-spectrum binding properties and high affinity for the cell wall-derived PAMPs, especially for LTA, DAP-PGN and LPS, according to the ordinate value, which represents the thickness of sensing probes. In comparison of the binding results of laminarin and mannan, which are two crucial fungal PAMPs, *Ap*-hemolin shows higher binding ability to mannan than to laminarin at the same concentration.

### Involvement of Ap-Hemolin in Antimicrobial Peptide Production

**Figure 4:**
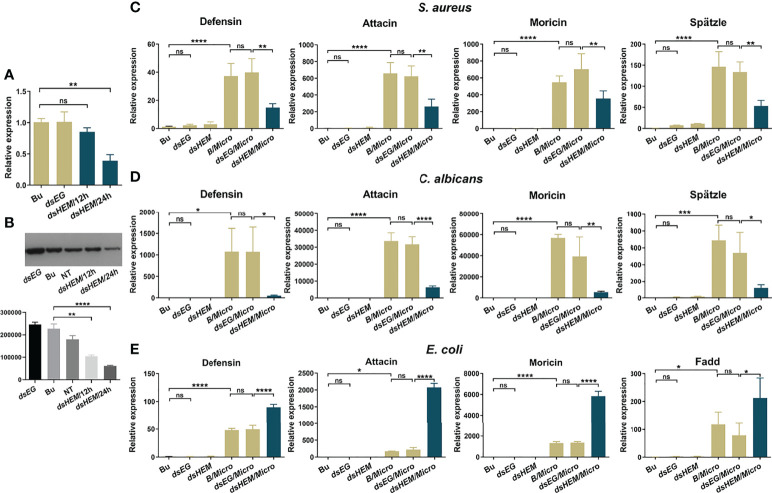
Effect of hemolin on the induction of antibacterial peptide synthesis in (*A*) *pernyi* larvae by qPCR analysis. RNAi efficiency within 24 hr post RNAi-treatment was estimation by qPCR **(A)** and western blotting analysis **(B)**. For qPCR, the whole body of larvae was collected at 12 and 24 hr post-injection with *dshemolin* (*dsHEM*) or *dsEGFP* (*dsEG*) double-stranded RNA. Larvae injected with buffer (Bu) were used as controls. For western blotting analysis, 10 μg of plasma samples from *dshemolin*-treated and control larvae were used. NT: native sample without treatment. The results represent 3 biological replicates and the gray-scale statistical was shown below. **(C-E)** Relative mRNA expression levels of antibacterial peptides and the representative factors in Toll or Imd signaling pathway in response to microbial stimulation. Micro: larvae collected 12 hr after injection with formaldehyde-killed microbial cells (10^5^ cells per larva); *dsEG*/*Micro*: larvae were first injected with *dsEGFP* for 24 hr and then immune-challenged with microbial cells for another 12 hr; *dsHEM*/*Micro*: larvae were first injected with *dshemolin* for 24 hr and then immune-challenged with microbes for another 12 hr. Microbes: *E. coli, S. aureus* and *C. albicans.* The relative expression level of antibacterial peptides and signaling pathway related factors detected by qPCR: defensin, attacin, moricin, spätzle and Fadd. Each bar represents the mean ± SD (N=3). ****: p<0.0001, ns, no significant difference.

As shown in **Figure 4A**, at 24 hr post-injection, the hemolin mRNA level of the *dshemolin* group displayed effective knockdown with a 65% decline. After *S. aureus* injection, considerable downregulation of the mRNA levels of all these effector molecules in the *Ap*-larvae was observed (**Figure 4C**). Similar but more remarkable results were also observed in the case of fungi *C. albicans* stimulation (**Figure 4D**). In contrast, hemolin knockdown followed by *E. coli* injection led to a significant increase in the transcript levels of three selected antimicrobial peptides (**Figure 4E**). To explore whether the regulation of hemolin on AMP production works through the Toll or Imd pathways, further investigation was performed simultaneously to evaluate the mRNA level of *spätzle* and *Fadd* in the *hemolin^-^* samples. Stirringly, the transcript level of spätzle in *S. aureus*- and *C. albicans*-triggered samples also declined when the expression of hemolin was knocked down, suggesting the regulation of hemolin on AMP synthesis might be *via* the Toll pathway. While the expression of *Fadd*, an important intracellular factor of Imd pathway, rose when the expression of hemolin was knocked down in the *E. coli* immune-challenged larvae. The expression trend of *Fadd* is consistent with that of AMPs detected in the same samples, implying the negative regulation of hemolin on Imd signaling pathway.

### Contribution of Ap-Hemolin to Prophenoloxidase Activation

**Figure 5:**
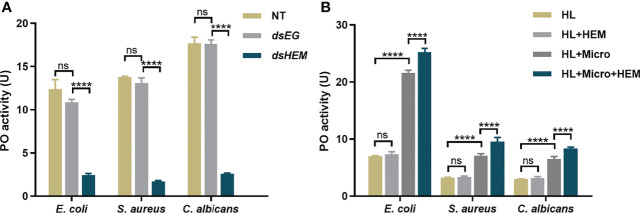
PO activity assay triggered by microbes. **(A)** The effects of endogenous hemolin on PPO cascade activation by microbes were investigated by monitoring the PO activity produced in hemolin knockdown plasma. Hemocyte-free plasma of 2^nd^ instar larvae was collected 24 hr after dsRNA injection. Then, the PO activity triggered by microbes was examined. *dsEG* and *dsHEM*: plasma samples from *dsEGFP*- and *dshemolin*-injected 2^nd^ instar larvae, respectively; NT: nontreated 2^nd^ instar larvae plasma. **(B)** The effects of exogenous hemolin on PPO activation stimulated by microbes were investigated with recombinant hemolin and 5^th^ instar native larvae plasma. HL: native cell-free hemolymph (total protein: 12 mg/ml); HEM: recombinant His_6_-*Ap*-hemolin (500 ng); Micro: *E. coli, S. aureus* and *C. albicans* (5 × 10^2^ cells per sample). Each bar represents the mean ± SD (N=3). ****: p<0.0001, ns, no significant difference.

**Figure 6:**
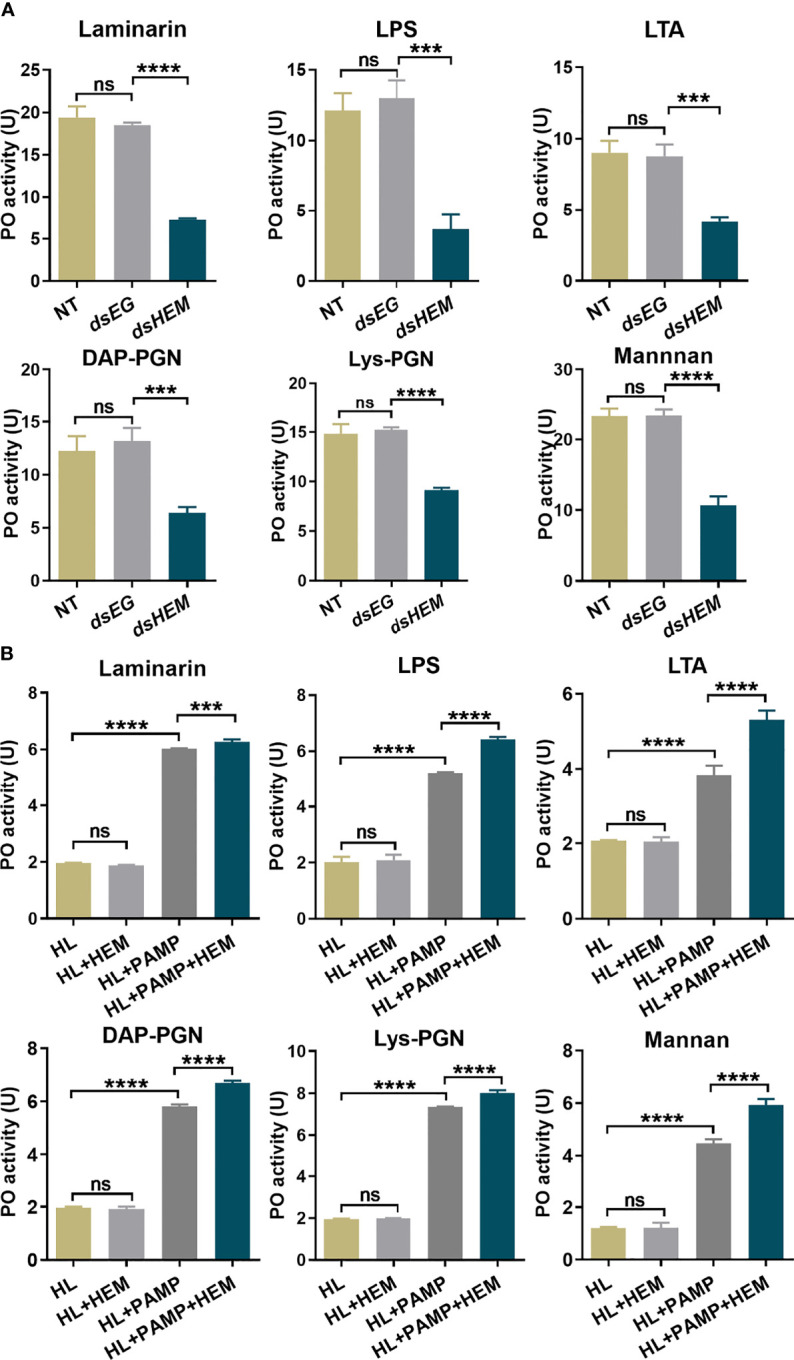
PO activity assay stimulated by soluble PAMPs. The effects of endogenous **(A)** and exogenous **(B)** hemolin on PPO cascade activation by soluble PAMPs were investigated by monitoring the PO activity produced in the *A. pernyi* larval plasma as described in **Figure 5**. PAMPs: laminarin (1 μg/ml), LPS (100 μg/ml), LTA (100 μg/ml), DAP-PGN (500 μg/ml), Lys-PGN (500 μg/ml) and mannan (1 mg/ml). *dsEG* and *dsHEM*: plasma samples from *dsEGFP*- and *dshemolin*-injected 2^nd^ instar larvae, respectively; NT: nontreated 2^nd^ instar larvae plasma. HL: native cell-free hemolymph (total protein: 12 μg/ml); HEM: recombinant His_6_-*Ap*-hemolin (500 ng). Each bar represents the mean ± SD (N=3). ***: p<0.001, ****: p<0.0001, ns, no significant difference.

**Figure 7:**
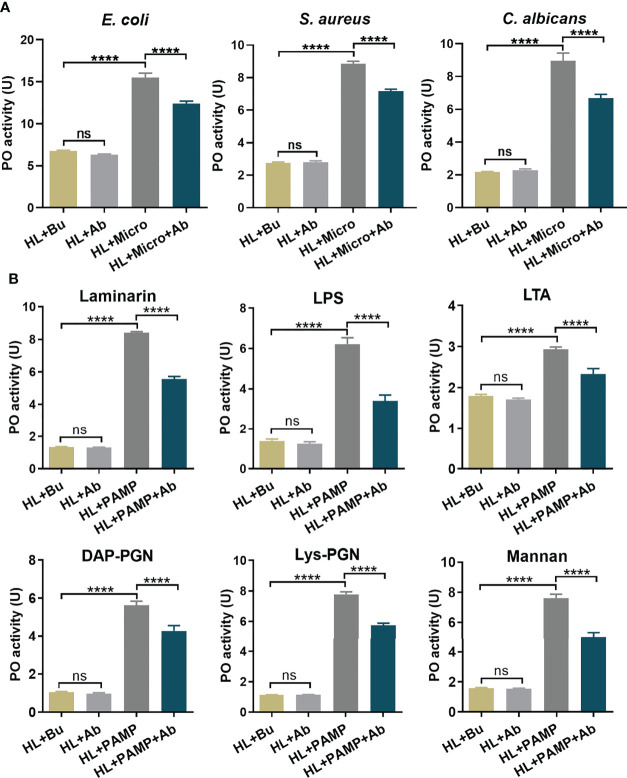
Pathogen dependent PO activation in *Ap*-hemolin blocked plasma. Hemocyte-free plasma samples were pre-incubated with anti-His_6_-*Ap*-hemolin antibody and 20 mM Tris-HCl, pH7.0, respectively. PO activity of these samples triggered by microbes **(A)** and soluble PAMPs **(B)** was examined and compared. HL: native cell-free hemolymph (total protein: 12 mg/ml); Bu: 20 mM Tris-HCl, pH7.0; Ab: anti-His_6_-hemolin antibody (0.8 mg/ml). Each bar represents the mean ± SD (N=3). ****: p<0.0001, ns, no significant difference.

To investigate the contribution of hemolin to the prophenoloxidase activation system, we treated and collected hemolin-deficient plasma by RNAi, and the knockdown efficiency of hemolin protein in hemolymph was examined by western blotting **(** **Figure S1****)**. For an equal protein amount in plasma, an approximate 75% substantial decline (gray-scale statistical analysis) in hemolin was detected after *dshemolin* injection compared to the negative controls (samples injected with *dsEGFP*, buffer and native plasma without treatment) at 24 hr post-injection (**Figure 4B**). Based on these results, hemocyte-free plasma from hemolin knockdown larvae was collected, and an *in vitro* PO activity assay was carried out. In comparison to nontreated or *dsEGFP*-treated plasma, significantly lower PO activity triggered by microbes in hemolin knockdown plasma could be observed (**Figure 5A**). Since *dsEGFP*-treated plasma showed no significant difference from native plasma, a deficiency of endogenous hemolin led to decreased PO activity in response to microbe stimulation, implying a positive effect of hemolin on PPO activation. To reconfirm the effect of *Ap*-hemolin on PPO activation acting as a PRR, an *in vitro* prophenoloxidase assay was carried out. The effect of various microbes on PPO activation in *A. pernyi* hemolymph was first estimated. Afterward, PPO activation was remarkably upregulated by the addition of the His_6_-*Ap*-hemolin/microbe mixture in comparison to samples with added microbes only (**Figure 5B**). Similar results of hemolin contributing to PPO activation in *A. pernyi* larval hemolymph were also detected when the stimulus was replaced by soluble PAMPs (**Figure 6**). On the other hand, the purified anti-His_6_-*Ap*-hemolin antibody was added into plasma to investigate the role of endogenous hemolin on PO activation in protein level. As shown in **Figure 7**, addition of elicitor led to prominent PO activity in plasma, but addition of antibody resulted in significantly lower PO activity than control plasma without antibody treatment. These results together suggesting that *Ap*-hemolin might contribute to up-regulation of PPO activation system.

## Discussion

The IgSF is the largest glycoprotein family with more than 700 gene members in the human genome, in which antibodies are the most well-known members that function as immune recognition molecules with high affinity and specificity to foreigners (37). Although no antibodies have been discovered in invertebrates (3), several IgSF members have been identified as cell adhesion molecules, such as fasciclin, neuroglian and Down syndrome cell adhesion molecule (Dscam), during the development of the insect nervous system instead of being involved in immune responses (38–40). Since it is known that IgSF existed prior to the evolutionary divergence of vertebrates and invertebrates, evolutionarily speaking, it remains possible that some proteins from IgSF could have an antibody-like role in immune surveillance in invertebrates. Hemolin is thus far the only invertebrate-derived IgSF member found to be upregulated upon infection. However, most previous studies revealed that the expression of hemolin in insect species exhibited different sensitivities to diverse intruders. For instance, in *Samia cynthia ricini* larvae, the expression of hemolin was strongly induced in fat bodies by an injection of bacterial cells (*Bacillus licheniformis* and *M. luteus*) or PGNs and was very weakly induced by *E. coli*, lipopolysaccharide or curdlan but was not changed by oligo chitin (14). The mRNA level of hemolin was significantly increased in response to bacterial stimulation but unaffected by natural baculoviral infection in the fat bodies and hemocytes of noctuid moths (17), while an opposite result was reported in bombycoid moths in which both bacterial and baculovirus infection led to upregulation of hemolin in *A. pernyi* (41). In addition to pathogen infection, hemolin was also present at a very low level in many vital organs, the concentration of which increased dramatically at metamorphosis stages in the absence of microbial challenge and regulated by hormones (19, 23, 24, 42). In the wandering 5^th^ instar larval stage of *Plodia interpunctella*, major expression of hemolin was detected in the epidermis, rather than in the fat body or gut, and the level was increased by feeding with RH-5992 (an ecdysteroid agonist) but reduced by treatment with KK-42 (an ecdysteroid inhibitor), suggesting that hemolin probably protected larvae from potential infection in the wandering stage (15). A similar result was obtained in *G. mellonella* in which a profound rise in hemolin expression without bacterial inoculation occurred in the silk glands of isolated abdomens when they were induced to pupate by a topical application of 20-hydroxyecdysone (16). Taken together, the data above underline the paramount importance of hemolin in insect defense against infectious threats throughout the lifecycle.

In this study, we aimed to explore the immune function of hemolin in *A. pernyi*. Based on the previous result that *Ap*-hemolin was constitutively expressed in the hemolymph, fat body, Malpighian tube, epidermis and midgut (41), further investigations were carried out to detect the expression level of hemolin in the 5^th^ larval stage in response to microbial injections by qPCR. The results revealed that *Ap*-hemolin is an immune-inducible protein. Comparing the increased degree of mRNA in different tissues after stimulation, the expression of hemolin was the most sensitive in the midgut, followed by the epidermis and hemocytes, but the change was the smallest in the Malpighian tube **(** **Figure 2****)**. In the first 3 hr post-injection, hemolin expression increased rapidly in the epidermis and fat body but barely changed in the Malpighian tube (**Figures 2A, D** and **C**). Moreover, there were two mRNA level peaks of hemolin in the Malpighian tube and fat body. Several explanations might account for these results. *A. pernyi* larvae feed on the leaves of Quercus (oak) plants, which increases the risk of encountering a large variety of microbes on the surfaces of plants. To combat such a potentially harmful environment, *A. pernyi* may have evolutionarily developed an efficient immune system in the intestinal system. Highly expressed hemolin in the midgut might function in intestinal immune responses. Moreover, the epidermis is the first line of defense in insects, and the upregulated expression level of hemolin indicates its participation in the early immune response against invading foreigners in the “border areas”. Fat bodies are equivalent to the mammalian liver, which encodes most immune molecules. Hemolin from the fat body might be transported to other organs through blood circulation, playing various immune functions. Moreover, two expression peaks of hemolin in the fat body suggest the involvement of hemolin in different stages of immune responses, for example, the early humoral and the later cellular immune responses. The Malpighian tube is the excretory system of insects at the end of the intestinal system, rarely encountering invaders in the early infectious stage so that the expression of hemolin here exhibited the lowest sensitivity to bacterial injection. However, it is not known why there are two expression peaks in the Malpighian tube facing immune invasion, especially the first peak appearing at 6 hr after pathogen induction.

To date, accumulating evidence has shown that hemolin binds to microbial cells and promotes bacterial agglutination in a calcium-dependent manner. Further studies demonstrate the function of hemolin as a PRR exhibiting specific affinity for LPS and LTA (12, 27, 28). Although the binding sites of hemolin to LPS and LTA have been well analyzed and the binding of LPS and hemolin in *M. sexta* was found to be decreased by peptidoglycan in a competitive binding assay, which suggests the interaction of hemolin with peptidoglycan (27), the definite pattern recognition property of hemolin is not fully understood. Here, the binding capacity of hemolin in *A. pernyi* to six classical infectious microbes (covering Gram-positive bacteria, Gram-negative bacteria and fungi) and three insoluble and six soluble bacterial cell wall-derived PAMPs was evaluated by western blotting and biolayer interferometry assays. The results confirmed that hemolin in *A. pernyi* functioned as a versatile PRR (**Figures 1B**, **3**). Hemolin in *A. pernyi* showed a higher affinity for LTA than for LPS, which coincided with the result of hemolin in *M. sexta* (27). Surprisingly, in the case of Lys-PGN, the association and dissociation processes were consistent with the other five PAMPs at concentrations of 50 and 100 μg/ml. When the concentration reached 150 μg/ml, abundant Lys-PGN rapidly dissociated, causing the amount of binding fraction to be even less than that at 50 μg/ml, although the binding amount was amazingly high in the association section. It is assumed that the binding capacity of hemolin to Lys-PGN is limited. Incubation with too much Lys-PGN and hemolin will result in a “hook effect”, which is often found in the interaction between antigen and antibody. Although we have thus gained some knowledge about the pattern recognition property of hemolin, the recognition process of pathogens by PRRs is much more complex than we thought. In the initial identification research of hemolin in *M. sexta*, apolipophorins and serine protease inhibitors (serpins) were also eluted from the bacteria incubated larval hemolymph (11), suggesting that more than one PRR, and even potential unidentified PRRs, is involved in the interaction with pathogens. In particular, apolipoprotein-III, which has been generally identified as a broad-spectrum PRR, also binds to LTA and LPS. In the pattern recognition process of the same PAMP, taking LTA as an example, it is unclear whether competition or synergy exists between hemolin and apolipoprotein III, which requires more complete characterization of the pattern recognition mechanism of hemolin to diverse microbes. Moreover, the result that hemolin in *A. pernyi* was inducible by the injection of nucleopolyhedrovirus (NPV), baculovirus or dsRNA (25, 41) suggests that hemolin might bind to viruses perhaps *via* the nucleic acid component, which needs to be verified in the future.

In addition to facilitating pathogen recognition, hemolin also functions as an opsonin in mediating the ability of hemocytes to engulf bacteria through phagocytosis and to form melanotic nodules, which is confirmed to be of vital importance in insect survival against bacterial infection (43). Although hemolin is widely considered to lack direct antibacterial activity (10, 44), few experimental data have been provided in previous studies. In this study, a bacteriostatic assay of His_6_-*Ap*-hemolin was carried out *in vitro* by monitoring the turbidity change of six typical pathogenic microbes in liquid culture medium (change in quantity). In comparison to the conventional state of microbial strains in fresh medium, the addition of antibiotics significantly suppressed their growth and proliferation at an extremely low concentration. However, in the presence of recombinant hemolin, all the test strains preserved growth and proliferation, exhibiting no significant difference from that of the control group on optical density. This observation affirms that *Ap*-hemolin has an inability to inhibit bacterial growth and proliferation **(** **Figure S2****)**. Additionally, it was proposed that prior infection of *M. sexta* larvae with the nonpathogenic bacterium *E. coli* elicits the upregulation of both hemolin and antibacterial peptide gene expression, providing the host with a high protective effect against invading pathogens (45, 46). This interesting result inspired us to further observe the effect of hemolin on the expression of antimicrobial peptides. Knockdown of the hemolin gene in *A. pernyi* markedly decreased the expression of the antibacterial peptides *defensin*, *attacin* and *moricin* in response to G^+^ bacteria (*S. aureus*) and fungi (*C. albicans*) stimulation (**Figures 4C, D**). To further investigate the effect of hemolin on the significant antimicrobial peptide synthesis signaling pathway, the mRNA level of the key factor, spätzle, of the Toll signaling pathway was also evaluated. As expected, the transcript level of spätzle in *S. aureus*- and *C. albicans*-triggered samples declined when the expression of hemolin was knocked down. In contrast, remarkably high upregulation of the expression of the same three antibacterial peptides was detected in the *dshemolin*-treated larvae post injection with the G^-^ bacterium *E. coli* **(** **Figure 4E****)**. Similarly, the mRNA level of Fadd, a vital intracellular component of Imd signaling pathway increased. Although numerous antimicrobial peptides have been identified in insects, most of them have not been studied for their antibacterial activity and synthetic pathways. It is well accepted that antimicrobial peptides are synthesized *via* the Toll, Imd, JAK/STAT, and Hippo signaling pathways, and the transcription of the same antimicrobial peptide sometimes might be regulated by more than one signaling pathway and other different factors (47–49). Here, the results that the antibacterial peptide mRNA level changes in synergy with that of spätzle or Fadd provide evidence that hemolin might be involved in the regulation of antimicrobial peptide production *via* the Toll and Imd signaling pathway in response to different microbial invasion. Combined with the pattern recognition features of hemolin, one testable hypothesis has been that after pattern recognition, the compound of hemolin and immune-elicitors might stimulate the hemolymphatic serine protease cascades, leading to the activation of transmembrane receptor Toll, which then initiates the intracellular transcription of AMPs. However, in the case of G^-^ bacterial stimulation, the expression level of hemolin was negatively correlated with that of antimicrobial peptides, suggesting that hemolin might be a negative regulator in other signaling pathways for antimicrobial peptide synthesis (e. g. Imd pathway) or that there are other factors together involved in the regulation of antimicrobial peptide synthesis, leading to the current results. At present, we do not know why hemolin shows such opposite regulation on the mRNA level of the same antimicrobial peptides when stimulated by different microorganisms, but it is assumed that hemolin acts like PGRP-LF in *Drosophila*, blocking the function of PGRP-LC, the main receptor of DAP-type gram-negative peptidoglycan upstream of the Imd pathway (50, 51). *Ap*-hemolin might compete with PGRP-LC for binding immune elicitors, which leads to the inhibition of the Imd signaling pathway and ultimately the reduction of antibacterial peptide synthesis. These hypotheses will be verified when further knowledge is obtained regarding the antibacterial activity of defensin, attacin and moricin and their synthetic signaling pathways in *A. pernyi* in the future.

On the other hand, we sought to investigate the function of hemolin in the PPO activating system in *A. pernyi* hemolymph, and the results confirmed the participation of hemolin in *A. pernyi* PPO activation was triggered by different irritants. The relationship between hemolin and melanization was unique found in *H. cecropia* (33). It was reported that when injecting dsRNA of the lepidopteran immune protein hemolin in pupae of *H. cecropia*, a significant reduction was observed in phenoloxidase activity after 24 h, but not after 72 h, the result of which is consistent with that of hemolin in *A. perny*i here. In addition to the stimulation of *E. coli*, we also detected the effect of another two types of microbes, G^+^ bacteria *S. aureus* and fungi *C. albicans*, together with six kinds of PAMPs on hemolymph PPO activation. As expected, hemolin-deficient *A. pernyi* hemolymph displayed lower sensitivity to microbes and PAMPs than the control hemolymph (**Figures 5A**, **6A**). However, there were a few differences. In **Figure 5A**, native *A. pernyi* larval hemolymph showed almost the same sensitivity among the three types of microbial stimulation in PPO activation at the same concentration. When the activator was replaced by soluble PAMP, the sensitivity of PPO activation in larval hemolymph changed. One explanation for the results we sought is that there are various types of PAMPs on the surface of each microorganism, and the amount of PAMPs differs. The level of PO activity in **Figure 5A** stimulated by microbes was the synthesized result of all the PAMPs on the surface of the microorganisms, which thus exhibited almost no significant difference between microbial elicitors. However, different sensitivities of PPO activation in the *A. pernyi* hemolymph were detected on PAMPs. To stimulate PO activity in a specific range (10-25 U), the additional amount of PAMPs differed. The results of **Figure 6A** revealed that the PPO in native larval hemolymph was activated by laminarin but difficult to be activated by mannan, which might be closely related to the species of pathogens that *A. pernyi* encounters in the long-term living environment. Additionally, hemolin-blocked plasma was also used to investigate the role of endogenous hemolin on PO activation at the protein level. Anti-hemolin polyclonal antibody was added to the plasma to block endogenous hemolin. Consistent with the results shown in **Figures 5A** and **6A**, the addition of the pathogen alone led to prominent PO activity in plasma, but the activation was significantly suppressed in the plasma preincubated with the anti-hemolin antibody (**Figure 7**). Furthermore, on the premise that *Ap-*hemolin is observed as a broad-spectrum pattern recognition receptor, PO activity is speculated to be associated with its pattern recognition property. To confirm this hypothesis, recombinant *Ap*-hemolin was prepared to investigate the effect of exogenous addition of hemolin on PO activity. The Ap-hemolin/elicitor (microbes or PAMPs) mixture increased PPO activation in the *A. pernyi* larval hemolymph, which was significantly higher than PAMP or hemolin alone (**Figures 5B**, **6B**). Since the native hemolymph contains all endogenous components needed for PPO cascade activation, Ap-hemolin itself failed to activate PPO active system, implying that combination of pathogens is necessary for hemolin contributing in PPO activation system. In addition, PO activation in hemolymph was detected to be pathogen-dependent within a certain concentration range when the amount of r*Ap*-hemolin was excessively added. The link between hemolin and PPO activation suggests that *Ap*-hemolin probably serves as a pattern recognition receptor and plays a crucial positive regulatory role in triggering the hemolymph PPO cascade in the defense against microbial infection in *Antheraea pernyi*.

In conclusion, we found that hemolin serves as a versatile pattern recognition receptor participating in the humoral immunity of *A. pernyi*, but there is still much to be further investigated.

## Data Availability Statement

The original contributions presented in the study are included in the article/**Supplementary Material**. Further inquiries can be directed to the corresponding authors.

## Author Contributions

All the experiments were performed under the guidance of XW and RZ. XH, TZ, YC and YL designed the study and contributed to data analysis and interpretation and drafting of the manuscript. SZ and JZ critically reviewed the analysis and made substantial contribution to the manuscript. All authors reviewed the manuscript and approved the final version.

## Funding

This work was financially supported by the National Natural Science Foundation of China (Grant numbers: 31772518 and 31970485); the Innovation Team Project and Talent Support Program of Liaoning Province Colleges and Universities (Grant number: LT2019013 and LR2019071); the Ph.D. Starting Foundation of Liaoning (Grant number: 2019-BS-230); the Innovation and Entrepreneurship Training Program for Students of Shenyang Pharmaceutical University (Grant number: YQ202115) and the Rolling Support Plan of Shenyang Pharmaceutical University for Youth Development (Grant numbers: ZQN2014A05).

## Conflict of Interest

Author YL is employed by Liaoning Applos Biotechnology Co. Ltd.

The remaining authors declare that the research was conducted in the absence of any commercial or financial relationships that could be construed as a potential conflict of interest.

## Publisher’s Note

All claims expressed in this article are solely those of the authors and do not necessarily represent those of their affiliated organizations, or those of the publisher, the editors and the reviewers. Any product that may be evaluated in this article, or claim that may be made by its manufacturer, is not guaranteed or endorsed by the publisher.
