# Supplementary material for: A Versatile Hemolin With Pattern Recognitional Contributions to the Humoral Immune Responses of the Chinese Oak Silkworm Antheraea pernyi
Source: Front Immunol. 2022 May 20;13:904862. doi: 10.3389/fimmu.2022.904862 (PMC9163686; doi:10.3389/fimmu.2022.904862)
Supplement: Supplementary file 1 [file DataSheet_1.docx]

**Supplemental Fig. S1. RNAi efficiency estimation by western blotting.**

For RNAi, 200 ng or 500 ng of *dshemolin* was injected into the hemocoel of each larva (five individuals for each set), and the larvae were reared for an additional 12, 24, 36 and 48 hr before hemolymph collection. Larvae injected with 200 ng *dsEGFP* for 24 hr (***dsEGFP* 200ng/24h**) and the same volume of insect saline (**Buffer**), together with native ones without treatment (**NT**) were used as controls. Knockdown efficiency was detected by western blotting. The decline level of hemolin in *A. pernyi* hemolymph was evaluated with the anti-His_6_-*Ap*-hemolin antibody (total protein in each lane: 10 μg). ***dsHemolin* 200ng/12h**: cell-free plasma of larvae collected 12hr post injection with 200ng *dshemolin* per larva. The other illustrations represent the similar meaning as above and the results were biologically replicated three times.

**Fig. S1**

**
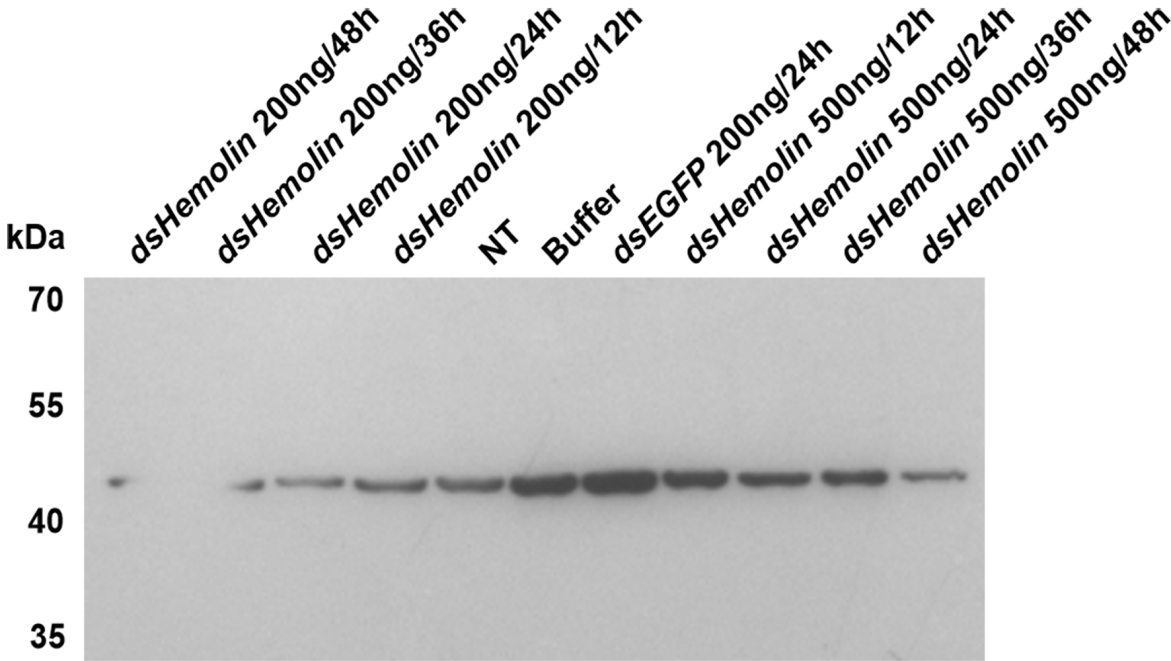
**
