# Supplementary material for: A Versatile Hemolin With Pattern Recognitional Contributions to the Humoral Immune Responses of the Chinese Oak Silkworm Antheraea pernyi
Source: Front Immunol. 2022 May 20;13:904862. doi: 10.3389/fimmu.2022.904862 (PMC9163686; doi:10.3389/fimmu.2022.904862)
Supplement: Supplementary file 2 [file DataSheet_2.docx]

**Supplemental Fig. S2. Microplate-based turbidimetric growth inhibition Assay.**

The growth inhibition properties of hemolin against microorganisms were monitored via the turbidity change of culture substances at OD_600_ (optical density at 600 nm). The test microorganisms used in this assay contained *E. coli*, *S. aureus*, *C. albicans*, *P. aeruginosa*, *M. luteus* and *S. cerevisiae*. The Luria-Bertani (LB) diluted the microorganisms into 5 × 10^2^ cells per sample. Antibiotics for each microorganism used in the assay were as followed: Kana: Kanamycin (50 ng/well), Amp: Ampicillin (60 ng/well), Flucz: Fluconazole (100 ng/well) and GM: Gentamicin (50 ng/well). Buffer (B): 20 mM Tris-HCl, pH7.0; HEM: recombinant His_6_-Ap-hemolin (20 μg/well). Each bar represents mean ± SD (N=3); analysis of variance with Tukey's multiple comparisons tests. *: p<0.05, **: p<0.01, ***: p<0.001, ****: p<0.0001, ns: no significant difference.

**Fig. S2**

**
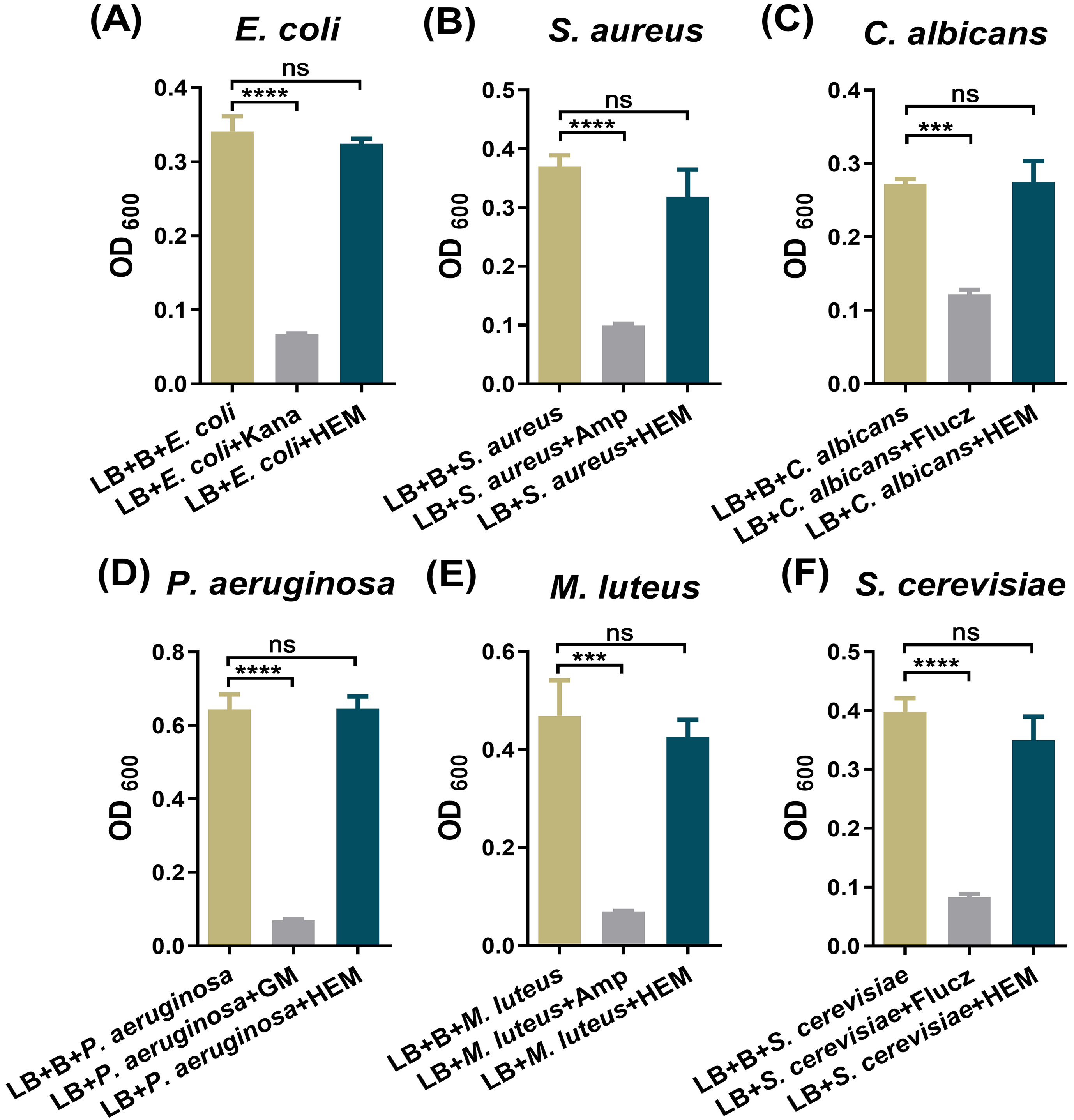
**
